# The *Campylobacter jejuni* Type VI Secretion System Enhances the Oxidative Stress Response and Host Colonization

**DOI:** 10.3389/fmicb.2019.02864

**Published:** 2019-12-17

**Authors:** Janie Liaw, Geunhye Hong, Cadi Davies, Abdi Elmi, Filip Sima, Alexandros Stratakos, Lavinia Stef, Ioan Pet, Abderrahman Hachani, Nicolae Corcionivoschi, Brendan W. Wren, Ozan Gundogdu, Nick Dorrell

**Affiliations:** ^1^Faculty of Infectious and Tropical Diseases, London School of Hygiene & Tropical Medicine, London, United Kingdom; ^2^Bacteriology Branch, Veterinary Sciences Division, Agri-Food and Biosciences Institute, Belfast, United Kingdom; ^3^Bioengineering of Animal Science Resources, Banat University of Agricultural Sciences and Veterinary Medicine – King Michael the I of Romania, Timisoara, Romania; ^4^The Peter Doherty Institute for Infection and Immunity, Department of Microbiology and Immunology, University of Melbourne, Melbourne, VIC, Australia

**Keywords:** campylobacter, type 6 secretion system, oxidative stress, chicken colonization, catalase, superoxide dismutase

## Abstract

The role of the Type VI secretion system (T6SS) in *Campylobacter jejuni* is poorly understood despite an increasing prevalence of the T6SS in recent *C. jejuni* isolates in humans and chickens. The T6SS is a contractile secretion machinery capable of delivering effectors that can play a role in host colonization and niche establishment. During host colonization, *C. jejuni* is exposed to oxidative stress in the host gastrointestinal tract, and in other bacteria the T6SS has been linked with the oxidative stress response. In this study, comparisons of whole genome sequences of a novel human isolate 488 with previously sequenced strains revealed a single highly conserved T6SS cluster shared between strains isolated from humans and chickens. The presence of a functional T6SS in the 488 wild-type strain is indicated by expression of T6SS genes and secretion of the effector TssD. Increased expression of oxidative stress response genes *katA*, *sodB*, and *ahpC*, and increased oxidative stress resistance in 488 wild-type strain suggest T6SS is associated with oxidative stress response. The role of the T6SS in interactions with host cells is explored using *in vitro* and *in vivo* models, and the presence of the T6SS is shown to increase *C. jejuni* cytotoxicity in the *Galleria mellonella* infection model. In biologically relevant models, the T6SS enhances *C. jejuni* interactions with and invasion of chicken primary intestinal cells and enhances the ability of *C. jejuni* to colonize chickens. This study demonstrates that the *C. jejuni* T6SS provides defense against oxidative stress and enhances host colonization, and highlights the importance of the T6SS during *in vivo* survival of T6SS-positive *C. jejuni* strains.

## Introduction

*Campylobacter jejuni*, a Gram-negative microaerophilic bacteria, is the leading cause of bacterial foodborne gastroenteritis worldwide. *C. jejuni* infection in humans can lead to diarrhea, vomiting, abdominal pain, fever, with symptoms generally appearing 2–5 days following exposure to an infectious dose as low as 500–900 bacteria (Robinson, 1981; Kaakoush et al., 2015). Disease presentation can vary depending on geographical region, with infections in low- and middle-income countries typically presenting with watery, non-inflammatory diarrhea whilst infections in high income countries display more severe disease, presenting with bloody inflammatory diarrhea (Coker et al., 2002). Campylobacteriosis is generally self-limiting, however, around 1 in 1,000 cases can develop severe auto-immune complications such as Guillain-Barré syndrome or Miller Fisher syndrome (Ang et al., 2001).

*Campylobacter jejuni* is most commonly transmitted through the handling and consumption of raw or undercooked poultry, but can also be spread through unpasteurized milk, contaminated water and cross contamination with other foods (Young et al., 2007; Kaakoush et al., 2015). *C. jejuni* colonizes chickens and other avian species and an estimated 70% of raw chicken sold in supermarkets in the United Kingdom will be contaminated with *C. jejuni* (Kaakoush et al., 2015). *C. jejuni* was previously regarded as a harmless commensal in the digestive tract of chickens, but recent studies indicate that colonization by *C. jejuni* is not asymptomatic, resulting in weight loss and slow growth of the infected poultry (Hermans et al., 2012; Wigley, 2015). The spread of *C. jejuni* through chicken flocks in farms can have a vast economic impact on the poultry industry and an increased spread of *C. jejuni* in chickens can subsequently affect the rates of infection in humans (Newell and Fearnley, 2003; Skarp et al., 2016).

During host colonization and infection, *C. jejuni* is exposed to conditions in the host gastrointestinal tract that present as physical and chemical stresses, including oxidative stress (Kim et al., 2015; Flint et al., 2016). Oxidative stress involves the generation of reactive oxygen species (ROS) that cause damage to nucleic acids, membranes and proteins of bacteria. In order to survive in this hostile environment, *C. jejuni* must defend against oxidative stress with enzymes that degrade ROS, such as SodB (superoxide dismutase), KatA (catalase), and AhpC (hydroperoxide reductase) (Kim et al., 2015). Regulation of the *C. jejuni* oxidative stress response is controlled by multiple regulatory mechanisms involving PerR, Fur and CosR to respond to fluctuating levels of ROS. Two MarR-type transcriptional regulators, RrpA and RrpB, also play a role in oxidative stress response regulation (Gundogdu et al., 2015).

The Type VI Secretion System (T6SS) is a contact-dependent secretion machinery capable of delivering effector proteins to both prokaryotic and eukaryotic cells. First identified in *Vibrio cholerae* and *Pseudomonas aeruginosa*, the T6SS has since been found to be present in 25% of Gram-negative bacterial species (Mougous et al., 2006; Pukatzki et al., 2006). The structure of the T6SS resembles an inverted bacteriophage tail with homologous components. The T6SS consists of 13 core components (TssA-TssM) and accessory proteins such as the T6SS-associated gene (Tag) proteins (see Table 1). A functioning T6SS complex requires essential components such as the baseplate (TssEFGK), a membrane-anchoring structure (TssJLM), a contractile sheath (TssBC) wrapped around a needle-like tube (TssD) and a puncture tip (TssI) further sharpened by a spike (TagD) (Cianfanelli et al., 2016). Composed of interlocking TssB and TssC components, the contractile sheath is responsible for producing enough energy to force the TssD needle-like structure through the inner membrane out into the extracellular space and puncture a host membrane to deliver effector proteins (Cianfanelli et al., 2016; Salih et al., 2018). The extended contractile sheath is broken down and the components recycled by the TssH ATPase for further sheath assembly (Kapitein et al., 2013).

**TABLE 1:** Components of the bacterial Type VI Secretion System.

**TSS system**	**Orthologs**	**Putative function/location**
TssA	VasJ/ImpA	Docks to membrane complex, recruits baseplate complex, initiates/coordinates polymerization of TssD with sheath
TssB	VipA/ImpB	Contractile sheath
TssC	VipB/ImpC	Contractile sheath
TssD	Hcp	Secreted tube, effector
TssE	HsiF	Baseplate
TssF	VasA/ImpG	Baseplate
TssG	VasB/ImpH	Baseplate
TssH	ClpV/VasG	AAA+ATPase, sheath recycling
TssI	VgrG	Expelled spike, effector
TssJ	VasD/Lip/SciN	Membrane complex
TssK	VasE/ImpJ	Baseplate
TssL	IcmH/DotU/VasF	Membrane complex
TssM	IcmF/VasK	Membrane complex
TagD	PAAR	Tip of expelled spike

Delivery of effector proteins by the T6SS to target cells can exert a number of effects including colonization and niche establishment (Kapitein and Mogk, 2014; Ma et al., 2014; Drebes Dorr and Blokesch, 2018). T6SS effectors can target and eliminate bacterial and fungal competitors and also affect eukaryotic host cells (Murdoch et al., 2011; Trunk et al., 2018). To prevent self-intoxication by effector proteins, bacteria with the T6SS possess immunity proteins to neutralize the effects of the toxins (Kirchberger et al., 2017; Ringel et al., 2017; Fitzsimons et al., 2018). In competition with prokaryotic targets, T6SS can act either defensively or offensively; for example, the T6SS of *V. cholerae* and *Serratia marcescens* are offensive, apparently firing constantly and indiscriminately into the surrounding space, whilst the defensive T6SS of *P. aeruginosa* reacts only when fired upon in a “tit-for-tat” response (Gerc et al., 2015). T6SS effectors can subvert host cell processes by manipulating the host cytoskeleton, hindering host defense mechanisms, modulating the host inflammatory response and modifying host membrane structure (Hachani et al., 2016).

The T6SS can also defend against the production of ROS through secretion of effectors. For example, the T6SS-4 of *Yersinia pseudotuberculosis* secretes the effector YezP, which is able to bind to and sequester zinc ions and protect the bacteria from the effects of oxidative stress (Wang et al., 2015). The T6SS-4 of *Burkholderia thailandensis* also secretes effectors TseM for the uptake of manganese ions and TseZ for the uptake of zinc ions to mitigate the effects of oxidative stress. Similarly, enterohemorrhagic *Escherichia coli* secretes a T6SS effector, KatN, which facilitates survival of the bacteria in macrophages through decreasing the level of intracellular ROS (Wan et al., 2017).

Studies examining the prevalence of the T6SS in *C. jejuni* in Asia and Europe found a large variation in prevalence between regions. Harrison et al. (2014) observed in 2014 that up to 70% of isolates from chickens and humans in Vietnam were positive for the T6SS, whilst approximately only 3% of similar isolates from the United Kingdom were T6SS-positive. A further study in 2015 examining chicken isolates in Spain found a prevalence of 14%, whilst another study found that 28.8% of chicken isolates in Northern Ireland were T6SS-positive (Corcionivoschi et al., 2015; Ugarte-Ruiz et al., 2015). Other *Campylobacter* species can also carry the T6SS; the same study in Northern Ireland observed that 56.1% of *C. coli* found in retail chickens were T6SS-positive (Corcionivoschi et al., 2015). However, the large variation in prevalence may be due to differences in sample sizes, sample sources and in detection methods. Recent data suggests that the T6SS is becoming increasingly prevalent in *C. jejuni* strains, with indications that T6SS-positive strains are becoming more prevalent than T6SS-negative strains infecting chickens in farms, on raw poultry in supermarkets and even in hospital patients in the United Kingdom (Carmel Kelly, Agri-Food and Biosciences Institute, Personal Communication). A recent study in 2018 examining the presence of *C. jejuni* in wild birds of Finland observed that 49% of western jackdaw isolates and 72% of mallard duck isolates were T6SS-positive (Kovanen et al., 2018). This emphasizes the need to understand the role of the T6SS in *C. jejuni* and to develop intervention strategies to combat the rise of T6SS-positive *C. jejuni* strains (Sima et al., 2018).

In contrast to *V. cholerae* and *P. aeruginosa*, the role of the T6SS in *C. jejuni* is poorly understood. To date, only one *C. jejuni* T6SS cluster expressing a single TssD has been identified, compared to *P. aeruginosa* and *Yersinia pseudotuberculosis* which both possess multiple T6SS clusters exhibiting different functions (Lertpiriyapong et al., 2012). The structure of the *C. jejuni* T6SS is yet to be solved and TssD is thus far the only effector protein identified to be secreted by the *C. jejuni* T6SS (Lertpiriyapong et al., 2012; Bleumink-Pluym et al., 2013). Previous studies have demonstrated the *C. jejuni* T6SS may be important in host cell adherence and invasion, colonization, survival in bile salts and contact-dependent cytotoxicity toward red blood cells (Lertpiriyapong et al., 2012; Bleumink-Pluym et al., 2013). A recent study examined the structure of the TssD effector in *C. jejuni* and found that TssD is cytotoxic toward HepG2 human liver carcinoma cells (Noreen et al., 2018). However, whether the T6SS plays a role in the survival and infection of *C. jejuni* in poultry, a primary reservoir in humans is still unknown.

In this study, we investigated the role of the *C. jejuni* T6SS through whole genome sequencing of a T6SS-positive 488 wild-type strain (a recent human isolate from Brazil). We also sequenced the T6SS-positive 43431 wild-type strain (a human isolate from Canada) (Penner et al., 1983). We constructed defined isogenic mutants for genes encoding the contractile sheath components TssBC and needle structure TssD in the 488 wild-type strain and performed *in vitro* and *in vivo* characterization experiments in biologically relevant models. Our findings indicate that a functional T6SS is present in the *C. jejuni* 488 wild-type strain and the presence of the T6SS is important in defending against oxidative stress and enhancing *in vivo* survival, potentially thereby allowing strains harboring the T6SS to colonize and dominate in specific niches within a host.

## Results

### Bioinformatic Analysis of T6SS Gene Clusters in *C. jejuni* 488 and Other T6SS-Positive Wild-Type Strains Reveals Synteny

**FIGURE 1:**
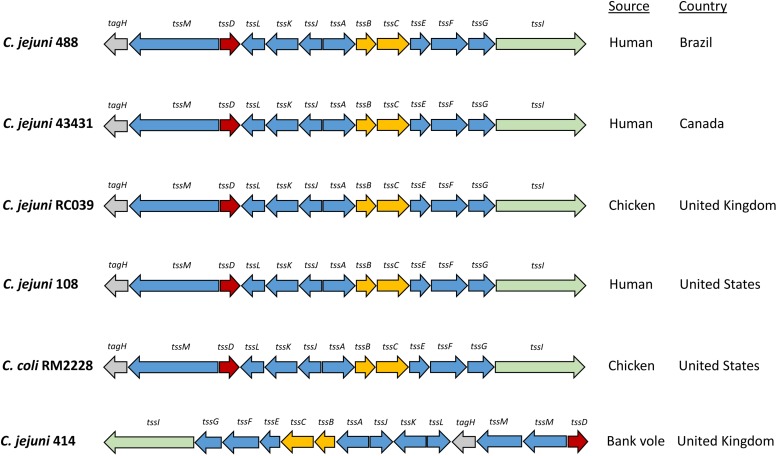
Type VI secretion system gene clusters of *C. jejuni* strains. The *C. jejuni* 488 and 43431 wild-type strains were sequenced using whole genome sequencing in this study. The genome sequence of *C. jejuni* RC039 was published by Corcionivoschi et al. (2015). The genome sequences for *C. jejuni* 108 and *C. coli* RM2228 was published by Bleumink-Pluym et al. (2013). The genome sequence for *C. jejuni* 414 was published by Lertpiriyapong et al. (2012).

Whole genome sequencing of *C. jejuni* 488 wild-type strain (a recent human isolate from Brazil) and *C. jejuni* wild-type strain 43431 (a human isolate from Canada) (Penner et al., 1983) was performed. *C. jejuni* wild-type strain RC039 (a chicken isolate from Northern Ireland) was previously sequenced (Corcionivoschi et al., 2015). The presence of a single T6SS cluster was observed in all three strains. All T6SS core components were identified, however, the TssH (ClpV) ATPase responsible for disassembly of the contracted sheath components is absent from the T6SS cluster of all *C. jejuni* strains sequenced thus far. The genome coordinates of the T6SS cluster in the *C. jejuni* 488 strain is listed in Supplementary Table S1. Comparisons of the 488, 43431, and RC039 genome sequences with previously published sequences for other T6SS-positive *C. jejuni* strains (Lertpiriyapong et al., 2012; Bleumink-Pluym et al., 2013) revealed a T6SS cluster that is highly conserved, sharing synteny between strains and also with other *Campylobacter* species isolated from humans and chickens (Figure 1). The 414 strain, isolated from wild bank voles in the United Kingdom, has a different gene arrangement in the T6SS cluster, but the same core components are still present.

### T6SS Genes *tssB*, *tssC tssD* Are Expressed in the 488 Wild-Type Strain

**FIGURE 2:**
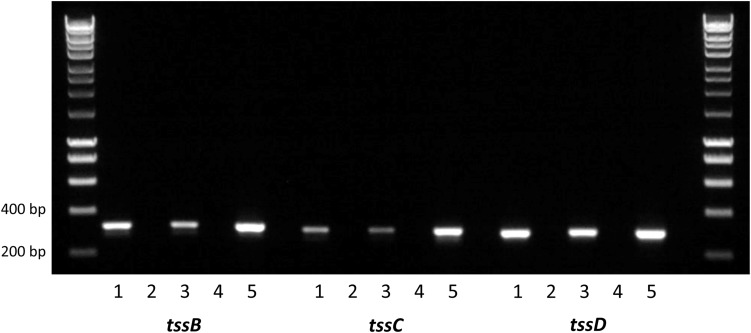
Reverse transcription PCR analysis of T6SS gene expression in the 488 wild-type strain. RNA was isolated from 488 wild-type strain. Plate cultures were grown on blood agar plates and incubated for 24 h under microaerobic conditions. Broth cultures were grown in Brucella broth for 16 h at 37°C with shaking at 75 rpm under microaerobic conditions. Extracted RNA was converted to cDNA. RT-PCR was performed using *tssB*, *tssC*, and *tssD* primers. The first and last lanes are the molecular weight marker (HyperLadder 1 kb, Bioline, United Kingdom). Lane 1: 488 cDNA (plate culture); Lane 2: negative control (488 cDNA from plate culture synthesized without reverse transcriptase); Lane 3: 488 cDNA (broth culture); Lane 4: negative control (488 cDNA from broth culture synthesized without reverse transcriptase); and Lane 5: positive control (488 gDNA). Expected sizes are 316 bp (*tssB*), 300 bp (*tssC*), and 287 bp (*tssD*).

Composed of interlocking TssB and TssC proteins, the contractile sheath is responsible for producing enough energy to force the TssD needle-like structure through the inner membrane out into the extracellular space and across host membranes (Cianfanelli et al., 2016). To investigate whether the *C. jejuni* 488 wild-type strain has a functional T6SS, the expression of the contractile sheath genes *tssB* and *tssC* as well as the *tssD* gene was investigated using RT-PCR. *tssB*, *tssC tssD* were all found to be expressed when the 488 wild-type strain is grown both in broth culture for 16 h and on blood agar for 24 h, indicating that the *C. jejuni* T6SS contractile sheath is produced under different growth conditions (Figure 2).

### *C. jejuni* 488 Wild-Type Strain Has a Functional T6SS That Secretes TssD

**FIGURE 3:**
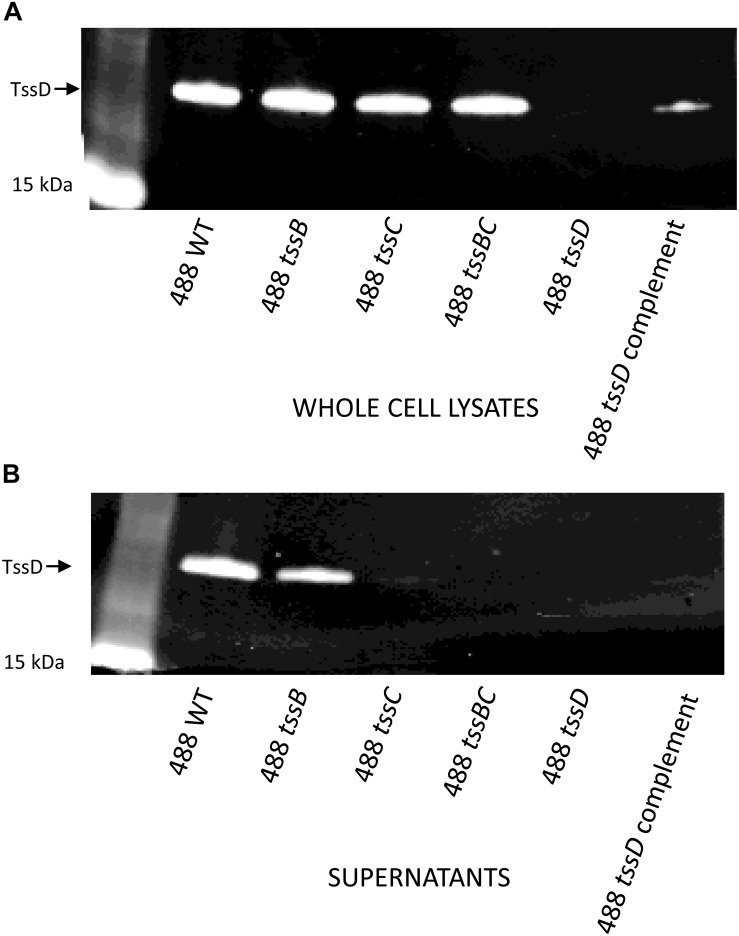
Detection of TssD in *C. jejuni* whole cell lysates and supernatants. **(A)** Detection of TssD in *C. jejuni* whole cell lysates. Whole cell lysate samples were prepared from 16 h cultures of *C. jejuni* 488 wild-type strain, 488 *tssB* mutant, 488 *tssC* mutant, 488 *tssBC* double mutant, 488 *tssD* mutant, and 488 *tssD* complement grown in Brucella broth. 13 μg of samples were loaded onto SDS-PAGE. Western blotting was performed using a TssD antibody, with a recombinant TssD protein used as a positive control. **(B)** Detection of TssD in *C. jejuni* supernatants. Bacterial supernatant samples were prepared from 16 h cultures of *C. jejuni* 488 wild-type strain, 488 *tssB* mutant, 488 *tssC* mutant and 488 *tssBC* double mutant grown in Brucella broth. Supernatant samples were concentrated via TCA precipitation and 30 μg of samples were loaded onto SDS-PAGE. Western blotting was performed using a *C. jejuni* TssD antibody. *C. jejuni* TssD has an estimated molecular weight of 18 kDa.

Previous studies have used the secretion of TssD as evidence that the T6SS is functional in *C. jejuni* (Lertpiriyapong et al., 2012; Bleumink-Pluym et al., 2013). In order to determine whether the T6SS in the 488 wild-type strain is functional, Western blotting was performed using a TssD antibody to detect the secretion of TssD. TssD is an approximately 18 kD protein that makes up the needle-like structure of the T6SS and has been shown in other bacteria to be important in the secretion of effector proteins (Cianfanelli et al., 2016). TssD is present in the whole cell lysate (Figure 3A) and also secreted into the supernatant of the 488 wild-type strain (Figure 3B), indicating that this strain has a functional T6SS. TssD is absent from both whole cell lysate and supernatant from the 488 *tssD* mutant.

### Inactivation of Individual Contractile Sheath Components Does Not Abolish *C. jejuni* T6SS Function

488 *tssB* and *tssC* single mutants were constructed to investigate whether knocking out a contractile sheath component would result in a non-functional T6SS. Inactivation of either *tssB* or *tssC* reduces but does not completely abolish secretion of TssD (Figure 3B), indicating that the absence of one of the contractile sheath components does not result in a completely non-functional T6SS in *C. jejuni*. A 488 *tssBC* double mutant was also constructed to test the hypothesis that function of the *C. jejuni* T6SS is only abolished in the absence of both contractile sheath components. As hypothesized, TssD was present in the whole cell lysate but absent from the supernatant of the 488 *tssBC* double mutant, thereby demonstrating that the *C. jejuni* T6SS is not functional when the entire contractile sheath structure is missing.

### T6SS in *C. jejuni* Is Associated With the Oxidative Stress Response

**FIGURE 4:**
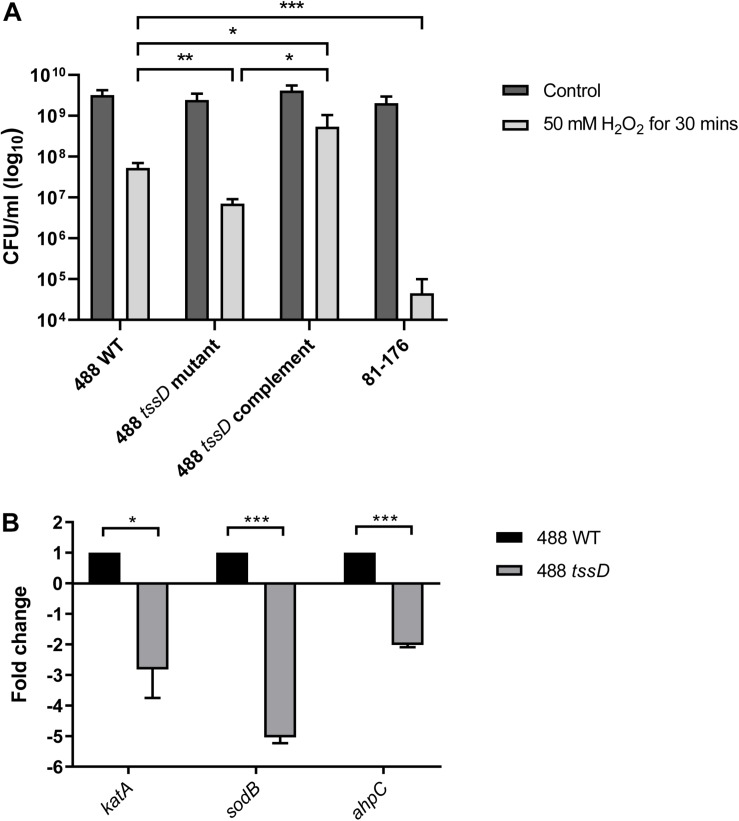
Type VI secretion system of *C. jejuni* is involved in the oxidative stress response. **(A)** Survival of 488 wild-type strain, 488 *tssD* mutant, 488 *tssD* complement, and 81–176 wild-type strain following exposure to oxidative stress. The strains were exposed to 50 mM H_2_O_2_ for 30 min, then serial dilutions were performed and CFUs/ml counted. Asterisks denote a statistically significant difference (^∗^*p* ≤ 0.05, ^∗∗^*p* ≤ 0.01, ^∗∗∗^*p* ≤ 0.001). **(B)** qRT-PCR analysis comparing expression of *katA*, *sodB*, and *ahpC* in the 488 wild-type strain and 488 *tssD* mutant. qRT-PCR analysis was performed using *katA*, *sodB*, and *ahpC* primers. Data was analyzed by the comparative CT method with *gyrA* housekeeping gene as the internal control. The relative expression of *katA*, *sodB*, and *ahpC* are shown. Asterisks denote a statistically significant difference (^∗^*p* ≤ 0.05, ^∗∗∗^*p* ≤ 0.001).

The T6SS has previously been linked with the oxidative stress response in *Y. pseudotuberculosis*, *B. thailandensis*, and enterohemorrhagic *E. coli* (Wang et al., 2015; Si et al., 2017; Wan et al., 2017). To investigate whether the *C. jejuni* T6SS is also associated with the oxidative stress response, the 488 wild-type strain, 488 *tssD* mutant and 81–176 wild-type strain were exposed to hydrogen peroxide (H_2_O_2_). Following 30 min exposure to 50 mM H_2_O_2_, the 488 wild-type strain exhibited significantly greater resistance to oxidative stress killing compared to the 488 *tssD* mutant and the 81–176 wild-type strain (Figure 4A). In order to investigate whether the increased susceptibility of the 488 *tssD* mutant to H_2_O_2_ is specific and not due to a potential membrane defect due to improper assembly of the T6SS in the bacterial membrane, antimicrobial susceptibility testing for the 488 wild-type strain, 488 tssD mutant and 488 tssD complement were performed. Disk diffusion assays were performed using ampicillin, amoxycillin/clavulanic acid, tetracycline and polymyxin B. A broth microdilution assay was performed using vancomycin. No differences in antimicrobial susceptibility were observed (Supplementary Table S2).

*katA*, *sodB*, and *ahpC* encode proteins involved directly in the breakdown of ROS. Expression of *katA*, *sodB*, and *ahpC* was investigated in the 488 wild-type strain and 488 *tssD* mutant using qRT-PCR. Expression of all three genes were significantly reduced in the 488 *tssD* mutant compared to the 488 wild-type strain. This data combined with the increased ability of the 488 wild-type strain to survive the effects of oxidative stress suggest that the T6SS is associated with the oxidative stress response in *C. jejuni* (Figure 4B).

### T6SS Increases *C. jejuni* Cytotoxicity in the *Galleria mellonella* Model of Infection

**FIGURE 5:**
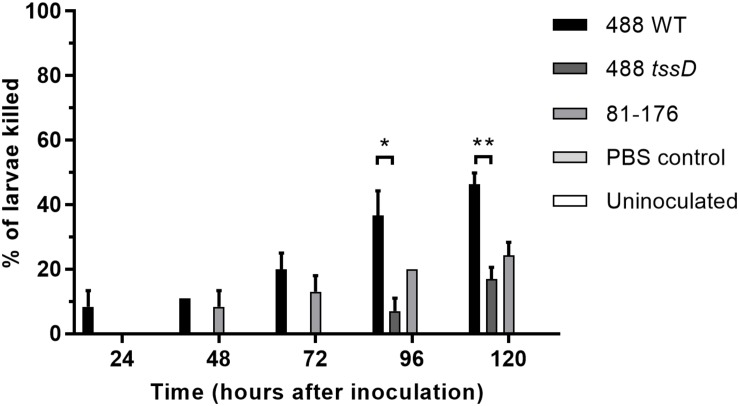
Infection of *Galleria mellonella* larvae with the 488 wild-type strain, 488 *tssD* mutant or 81–176 wild-type strain. Ten *G. mellonella* larvae were injected per bacterial strain with a syringe in the right foremost leg. Controls injected with PBS only were included along with uninoculated controls. Larvae were incubated at 37°C with counts of dead larvae recorded every 24 h over a period of 5 days. Asterisks denote a statistically significant difference (^∗^*p* ≤ 0.05, ^∗∗^*p* ≤ 0.01).

The larvae of *G. mellonella* (Greater wax moth) are an established model to study the pathogenesis of *C. jejuni* (Senior et al., 2011). In order to investigate whether the presence of a T6SS in *C. jejuni* enhances bacterial cytotoxicity, *G. mellonella* larvae were injected with *C. jejuni* strains and larvae survival examined over a duration of 5 days. Only the T6SS-positive 488 wild-type strain was cytotoxic to *G. mellonella* larvae after 24 h post-injection (Figure 5). The T6SS-negative 81–176 wild-type strain also exhibited some cytotoxicity, but at a lower level compared to the 488 wild-type strain after 48 and 72 h. After 96 and 120 h, cytotoxicity of the 488 *tssD* mutant was also observed but at a significantly lower level than the 488 wild-type strain.

### T6SS Enhances Both *C. jejuni* Interactions With and Invasion of Chicken Primary Intestinal Cells

**FIGURE 6:**
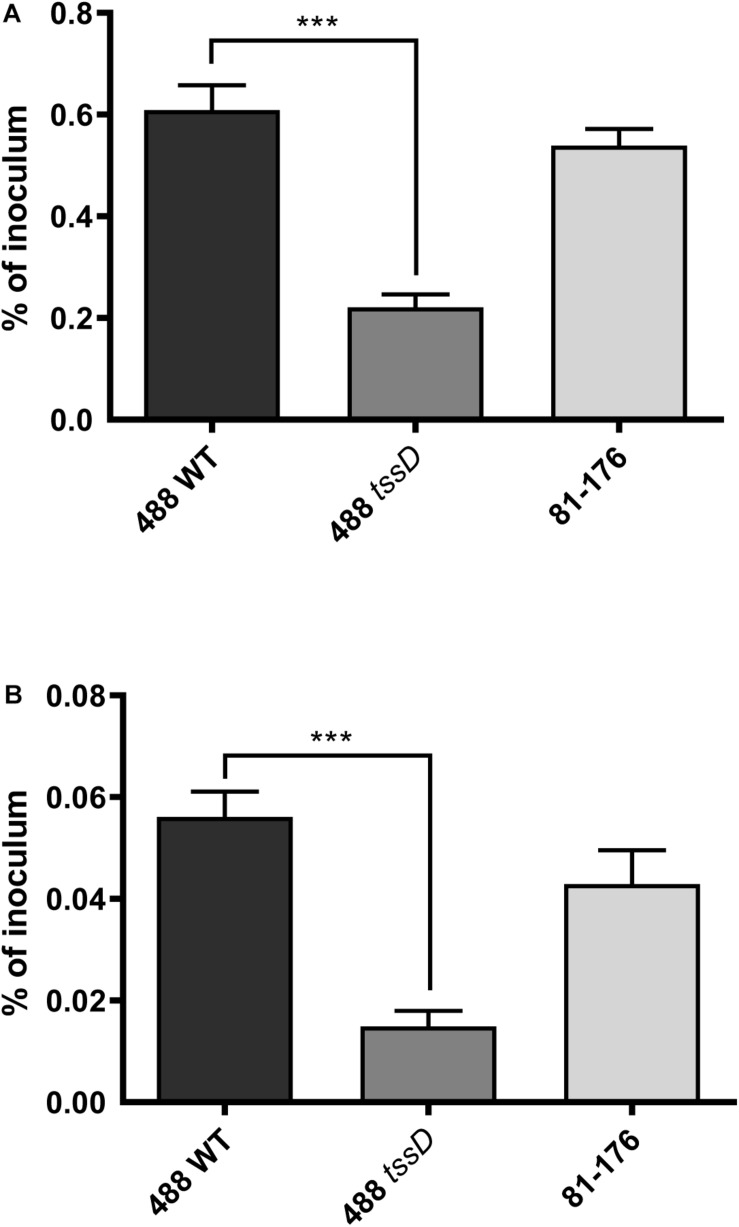
Interactions with and invasion of primary chicken intestinal cells with the 488 wild-type strain, 488 *tssD* mutant or 81–176 wild-type strain. Primary chicken intestinal cells were infected with *C. jejuni* strains at a MOI of 1,000:1. **(A)** For interaction assays, infected monolayers were washed with PBS and treated with 0.1% (v/v) Triton X-100. **(B)** For invasion assays, infected monolayers were washed with PBS and treated with gentamicin (400 μg/ml) for 2 h at 37°C. Cells were then washed with PBS and treated with 0.1% (v/v) Triton X-100. For both experiments, serial dilutions were then performed and the CFUs/ml counted. Asterisks denote a statistically significant difference (^∗∗∗^*p* ≤ 0.001).

Recent studies suggest that the prevalence of T6SS-positive *C. jejuni* strains is on the rise in high-income countries and there are indications that T6SS-positive strains are now prevalent over T6SS-negative strains infecting chickens in farms, on raw chicken in supermarkets and in hospital patients (Ugarte-Ruiz et al., 2015; Sima et al., 2018). To investigate whether the presence of the T6SS plays a role in the ability of *C. jejuni* to infect chickens, an *in vitro* model was used to examine the potential of *C. jejuni* to adhere to and invade chicken primary intestinal cells. The 488 wild-type strain exhibited significantly higher levels of adherence and invasion than the 488 *tssD* mutant. The T6SS-negative 81–176 wild-type strain also exhibited lower levels of adherence and invasion than the 488 wild-type strain, however, these differences were not significant (Figure 6).

### T6SS Enhances the Ability of *C. jejuni* to Infect Chickens

**FIGURE 7:**
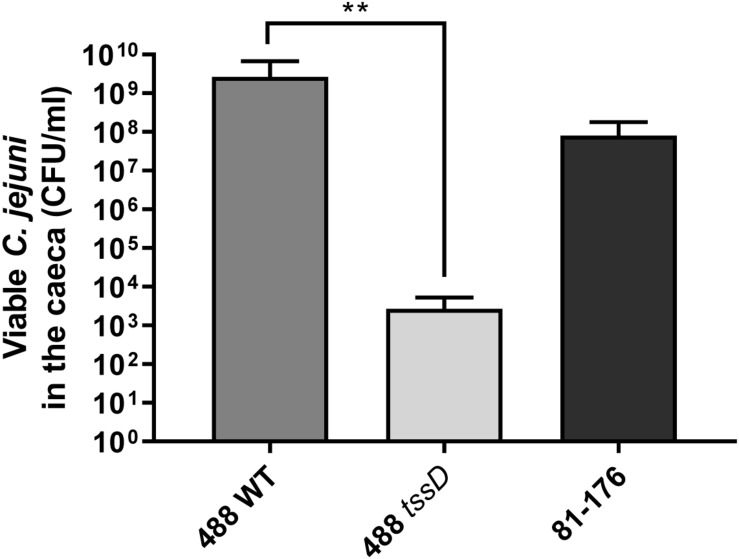
Infection of broiler chickens with the 488 wild-type strain, 488 *tssD* mutant or 81–176 wild-type strain. Ten 15-day old Ross 308 broiler chickens were inoculated with approximately 1 × 10^8^ CFU/ml of either the 488 wild-type strain, the 488 *tssD* mutant or the 81–176 wild-type strain. Chickens were euthanized 3 days post-infection. Caecum contents were plated and counts of viable *C. jejuni* in the samples performed. Asterisks denote a statistically significant difference (^∗∗^*p* ≤ 0.01).

To further investigate whether the presence of the T6SS plays a role in the ability of *C. jejuni* to infect chickens, chicken infection studies were performed. An identical pattern was observed after *in vivo* infection of 15-day old Ross 308 broiler chickens, where at 3 days post-infection the numbers of the 488 wild-type strain detected in the caeca were significantly higher than the numbers of the 488 *tssD* mutant or 81–176 wild-type strain (Figure 7). These results indicate that the presence of the T6SS is important in enhancing the ability of *C. jejuni* to colonize chickens.

## Discussion

The *C. jejuni* T6SS is a functional secretory mechanism that appears distinct from the well-elucidated *P. aeruginosa* and *V. cholerae* T6SS model systems. Whole genome sequencing of a novel 488 strain and the T6SS-positive 43431 strain revealed a single T6SS cluster containing one copy of the *tssD* gene that encodes the needle-like tube of the T6SS, but the absence of *tssH* which encodes the ATPase that breaks down and recycles the TssBC contractile sheath. This is in contrast to the T6SS model systems of *P. aeruginosa* and *V. cholerae* where multiple T6SS clusters are present with differing functions. The absence of a *tssH* ortholog had previously been observed in other *C. jejuni* strains in other studies (Lertpiriyapong et al., 2012; Bleumink-Pluym et al., 2013). *Burkholderia* species, *Helicobacter hepaticus*, *Francisella tularensis*, and *Salmonella enterica* also appear to lack a TssH component. Despite the absence of TssH, the presence of a functional T6SS in all these organisms including *C. jejuni* suggests there may be alternate mechanisms for contractile sheath recycling. In *V. cholerae* a study demonstrated that whilst TssH is not essential, it is important in increasing the efficiency of the T6SS in inter-bacterial competition assays (Bachmann et al., 2015).

Comparison of the *C. jejuni* strains sequenced in this study with previously published sequences of other *C. jejuni* strains as well as other *Campylobacter* species revealed a T6SS cluster that is very closely conserved with all core genes present in the same arrangement. *C. jejuni* 488 (human isolate from Brazil), 43431 (human isolate from Canada), 108 (human isolate from the United States), RC039 (chicken isolate from the United Kingdom), and even *C. coli* RM2228 (chicken isolate from the United States) all share the same conserved T6SS cluster. Only *C. jejuni* 414 had a different gene arrangement in the T6SS cluster. 414 is an environmental strain, isolated from a wild bank vole in the United Kingdom (Williams et al., 2010). It is possible that agricultural intensification practices which readily facilitate passing of strains between chickens and humans could lead to these strains to share more of a conserved T6SS compared to the 414 environmental strain.

Further investigations indicated that the novel 488 strain has a functional T6SS capable of secreting the TssD effector, corroborating previous studies which have shown TssD to be secreted from 43431 and 108 strains (Lertpiriyapong et al., 2012; Bleumink-Pluym et al., 2013). However, the contractile sheath components TssBC and their function had not previously been studied in *C. jejuni*. In other bacteria, an intact contractile sheath structure is important for a fully functioning T6SS as removal of either TssB/VipA or TssC/VipB from the contractile sheaths described in *V. cholerae* result in a defective T6SS unable to secrete effectors (Basler et al., 2012; Kudryashev et al., 2015; Brackmann et al., 2018). In this study we have shown that both contractile sheath genes are expressed when the 488 strain is cultured in broth and on solid plates. Isogenic 488 *tssB* and *tssC* mutants were constructed and shown to still be able to secrete TssD at a reduced level compared to the wild-type strain. The observation that the *C. jejuni* T6SS does not require both TssB and TssC components to be present for the T6SS to be able to secrete TssD is unusual and differs from previous observations of the T6SS in *V. cholerae*. This suggests that in *C. jejuni*, as long as either TssB or TssC is present, the T6SS remains capable of secreting TssD at a reduced level. However, this does not suggest that the contractile sheath components in *C. jejuni* may differ from those in the T6SS model systems of *P. aeruginosa* and *V. cholerae*, rather that the contractile sheath components may be interchangeable or be able to compensate for the absence of the other component via a yet unknown process.

The importance of the T6SS in countering the effects of oxidative stress has previously been shown in *Y. pseudotuberculosis*, *B. thailandensis*, and enterohemorrhagic *E. coli* (Wang et al., 2015; Si et al., 2017; Wan et al., 2017). In this study, we show that the *C. jejuni* T6SS also appears to play a role, with the genes that encode KatA, SodB, and AhpC that are involved directly in the breakdown of ROS all expressed at significantly higher levels in the 488 wild-type strain compared to the 488 *tssD* mutant. The 488 wild-type strain with an intact T6SS is also more resistant to the effects of oxidative stress compared to the 488 *tssD* mutant. We speculate that TssD positively regulates the expression of genes involved in the breakdown of ROS, and in turn this results in greater resistance to oxidative stress in strains harboring an intact T6SS cluster.

A MarR family transcriptional regulator TctR was recently shown to regulate the T6SS-2 gene cluster in *B. pseudomallei* (Losada et al., 2018). Previous studies have also shown that the MarR family transcriptional regulators RrpA and RrpB are important in regulating both oxidative and aerobic stress responses in *C. jejuni* and therefore play a role in enhancing bacterial survival both in the hosts and in the environment (Gundogdu et al., 2011, 2015, 2016). As our results indicate that the *C. jejuni* T6SS is also associated with the oxidative stress response, it is tempting to speculate that the RrpA and RrpB regulators may in some way be linked to the T6SS. Further studies will be required to investigate this hypothetical link.

Based on our data we propose that presence of the T6SS cluster in *C. jejuni* strains also confers a competitive advantage to these strains within a host. The T6SS-positive 488 wild-type strain is more cytotoxic in the *G. mellonella* model than the T6SS-negative 81–176 wild-type strain and the 488 *tssD* mutant. This suggests that the presence of a T6SS increases the cytotoxicity of *C. jejuni* in the *G. mellonella* model of infection and secreted T6SS effectors may also be important in causing cytotoxicity to other organisms. The 81–176 strain was selected as a negative control in this study due to the absence of the T6SS in this strain. Despite lacking the T6SS, 81–176 is a hypervirulent strain capable of causing severe disease and harbors two plasmids, pTet and pVir, that play a role in increased virulence (Black et al., 1988; Bacon et al., 2000). Therefore, any differences observed between 488 and 81–176 could potentially be due to a diversity of virulence factors in these two strains rather than the presence or absence of the T6SS.

This study is the first to examine the role of T6SS of *C. jejuni* in a biologically relevant model. Lertpiriyapong et al. (2012) utilized a murine model and found that a T6SS-positive strain to be more interactive with and invasive in RAW 264.7 macrophage cells and have a higher colonization potential in IL-10-deficient mice. However, it has previously been shown that *C. jejuni* does not colonize mice in the same manner as in chickens, as *C. jejuni* colonizes mice at a significantly slower rate than chickens and some strains such as 81–176 do not appear to colonize mice at all (Wilson et al., 2010). The *C. jejuni* 488 wild-type strain exhibits higher levels of adherence to and invasion of chicken cells and is also able to infect chickens at a much higher rate that the *tssD* mutant. The results indicate that the ability of *C. jejuni* to infect chickens is enhanced by the presence of the T6SS and the T6SS may be important as a colonization factor in the natural host of *C. jejuni*. The increased ability of *C. jejuni* strains with the T6SS to infect and colonize chickens is a particular concern for the agricultural and food industries tasked with reducing *C. jejuni* load in both live chickens on farms and on raw chicken meat in the supermarket (Sima et al., 2018). From a public health perspective, the rise of T6SS in *C. jejuni* is also problematic as there are indications that strains with the T6SS may cause more severe disease in humans (Harrison et al., 2014).

In summary, in this study we confirmed the presence of a single T6SS cluster among several *C. jejuni* strains and confirmed that the functional T6SS is capable of secreting the TssD effector. Our results indicated that the *C. jejuni* T6SS is involved in the oxidative stress response. Using *in vitro* and *in vivo* models we demonstrated the increased ability of T6SS-positive *C. jejuni* to colonize the natural avian host. Our findings highlight the importance of further understanding the function of the T6SS present in an expanding population of *C. jejuni*, the potential importance of the T6SS in colonization and niche establishment in different hosts and the potential for T6SS-positive *C. jejuni* strains to cause more severe disease in both chickens and humans.

## Materials and Methods

### Bacterial Strains and Growth Conditions

*Campylobacter jejuni* strains used in this study are listed in Supplementary Table S3. *C. jejuni* strains were grown at 37°C under microaerobic conditions (85% N_2_, 15% CO_2_ 5% O_2_) in a variable atmosphere chamber (Don Whitley Scientific, United Kingdom). Unless otherwise stated, *C. jejuni* were grown either on Columbia agar with 7% (v/v) horse blood (TCS Biosciences, United Kingdom) with the addition of Skirrow *Campylobacter* selective supplement or in Brucella broth (BD Diagnostics, United Kingdom). *E. coli* strains listed in Supplementary Table S4 were grown on lysogeny broth (LB) agar plates or in LB broth at 37°C. The appropriate antibiotics were added as required at concentrations of 50 μg/ml kanamycin, 100 μg/ml ampicillin or 10 μg/ml chloramphenicol for *C. jejuni* growth, with the concentration of chloramphenicol for *E. coli* growth increased to 50 μg/ml. All reagents were obtained from Oxoid (United Kingdom) unless otherwise stated.

### Whole Genome Sequencing

Genome sequencing of the *C. jejuni* 488 and 43431 (Poly et al., 2004) wild-type strains was performed as previously described by Ugarte-Ruiz et al. (2014). Briefly, sequencing was performed using the Illumina MiSeq 2 × 301 bp paired-end sequencing. To analyze the data quality, FastQC was used (Simon, 2010) followed by read trimming using Trimmomatic (v0.32) (leading’ and “trailing” setting of 3, a “sliding window” setting of 4:20 and a “minlength” of 36 nucleotides) (Bolger et al., 2014). Reads were mapped using BWA-MEM (v0.7.7-r441) against the genome sequence of *C. jejuni* NCTC 11168 (AL111168) (Li and Durbin, 2009). Assembly on unmapped regions was performed using Velvet Optimizer (v2.2.5) using n50 optimization (Zerbino and Birney, 2008; Gladman and Seemann, 2012). Contigs were ordered against *C. jejuni* NCTC 11168 (AL111168) strains using ABACAS (v1.3.1) (Assefa et al., 2009). Genome annotation was performed using Prokka (Seemann, 2014). Genomes were visualized using Artemis and ACT software (Carver et al., 2012). T6SS ORFs were identified using BLAST (Altschul et al., 1990; Gish and States, 1993). The 488 and 43431 genome sequences were uploaded to the EBI ENA database (Accession number PRJEB31331).

### Reverse Transcription PCR (RT-PCR) and Quantitative Real-Time PCR (qRT-PCR) Analyses

Total RNA was isolated using PureLink RNA Mini Kit (Thermo Fisher Scientific) from *C. jejuni* cultures grown for 16 h at 37°C with shaking at 75 rpm under microaerobic conditions in Brucella broth. TURBO DNA-free kit (Thermo Fisher Scientific) was used to remove DNA contamination and RNA was converted to cDNA using the SuperScript III First-Strand Synthesis System (Thermo Fisher Scientific). Reverse transcription PCR (RT-PCR) was performed according to the manufacturer’s instructions with primers listed in Supplementary Table S5. Quantitative real-time PCR (qRT-PCR) was performed using primers listed in Supplementary Table S5, with SYBR Green Master Mix (Applied Biosystems, United Kingdom) on the ABI 7500 Real-Time PCR System machine (Applied Biosystems). qRT-PCR data was analyzed by the comparative C_*T*_ method with *gyrA* housekeeping gene as an internal control (Ritz et al., 2002; Schmittgen and Livak, 2008).

### Construction of *tssB*, *tssC*, *tssD* Mutants and *tssBC* Double Mutant

*Campylobacter jejuni* mutants were constructed as previously described (Gundogdu et al., 2011). Briefly, the appropriate gene specific primers (see Supplementary Table S5) were used to amplify the gene of interest from *C. jejuni* genomic DNA. PCR products were cloned into a pGEM-T Easy vector (Promega, United Kingdom) and transformed into *E. coli* XL2-Blue if the unique restriction site was *Bam*HI or *Bgl*II. *E. coli* SCS110 were used if the restriction site was *Bcl*I. If none of these sites were present, inverse PCR mutagenesis was used to introduce a *Bgl*II site (Gundogdu et al., 2015). Plasmid DNA was digested with *Bam*HI, *Bcl*I, or *Bgl*II, a kanamycin or chloramphenicol resistance cassette was inserted and the resulting construct transformed into *E. coli*. Successful constructs were transformed into *C. jejuni* by electroporation and positive clones were confirmed by PCR and Sanger sequencing. For construction of a *C. jejuni tssBC* double mutant, the plasmid DNA construct used for the *C. jejuni tssC* mutant was transformed into *C. jejuni tssB* mutant by electroporation.

### Preparation of Whole Cell Lysates and Supernatants for Protein Secretion Assays

*Campylobacter jejuni* strains from 24 h plate cultures were inoculated to OD_600_ 0.1 in Brucella broth and incubated at 37°C with shaking at 75 rpm under microaerobic conditions until late log phase. The broth cultures were centrifuged at 4,000 rpm at 4°C for 30 min to separate the pellet and the supernatant.

To prepare a whole cell lysate sample, a pellet was re-suspended in PBS and sonicated for 10 min on high setting using a Bioruptor (Diagenode, Belgium). Sonicated samples were centrifuged at 13,000 rpm for 5 min and the resulting supernatant was added to 2X Laemmli sample buffer (Sigma-Aldrich, United Kingdom) then boiled at 95°C for 10 min, followed by centrifugation at 13,000 rpm for 5 min. To prepare a supernatant sample, supernatant was filtered through a 0.2 μm-pore-size filter (Millipore, United Kingdom) to remove remaining cells and the titrate was concentrated using an Amicon Ultra-15 centrifugal filter (10 kDa) (Millipore). Trichloroacetic acid (TCA) precipitation with acetone washes was performed to further concentrate the samples as described previously (Link and LaBaer, 2011). Following acetone washes, the pellet was re-suspended in 2X Laemmli sample buffer and boiled at 95°C for 10 min, followed by centrifugation at 13,000 rpm for 5 min.

### SDS-PAGE and Western Blot Analysis

BCA assay was performed to determine protein concentration in samples. 13 μg of whole cell lysate samples and 30 μg of supernatant samples were loaded along with Pageruler Plus Pre-stained Protein Ladder (Thermo Fisher Scientific, United Kingdom) onto NuPAGE Bis-Tris gel (12%) (Thermo Fisher Scientific) with MOPS running buffer (Thermo Fisher Scientific). Membrane transfer was performed using the iBlot 2 Dry Blotting System (Thermo Fisher Scientific). The membrane was blocked with 2% (w/v) milk overnight at 4°C, incubated with the TssD antibody in phosphate buffered saline with 0.1% (v/v) Tween-20 (PBS-T) for 1 h at room temperature, washed with PBS-T then incubated with the secondary goat anti-rabbit antibody in PBS-T for 1 h at room temperature. The membrane was washed twice with PBS-T, once with PBS scanned using the Odyssey Imaging System (LI-COR Biosciences, United Kingdom).

### TssD Antibody Production

The polyclonal anti-TssD antibody was produced by Capra Science Antibodies AB (Sweden). The *tssD* gene cloned into an expression vector and recombinant TssD protein was isolated and purified. For production of the TssD antiserum, purified TssD was immunized into a rabbit. Two boosts with the antigen were performed, followed by the first bleed. A further boost with the antigen was performed followed by the second bleed; the process was repeated for a third bleed. Antiserum collected from the third bleed was affinity purified using a peptide-coupled column for the anti-TssD antibody.

### Oxidative Stress Assay

*Campylobacter jejuni* from 24 h plate cultures were re-suspended in PBS, the OD_600_ was measured and the suspension adjusted with PBS to OD_600_ 1.0. Bacterial suspensions were exposed to 50 mM H_2_O_2_ for 30 min at 37°C stationary under microaerobic conditions. Serial dilutions were performed and dilutions plated onto blood agar plates. Plates were incubated at 37°C under microaerobic conditions and colonies counted after 48 h.

### Antimicrobial Susceptibility Assays

The disk diffusion assays were performed with ampicillin (10 μg), amoxycillin/clavulanic acid (2:1, 30 μg), tetracycline (30 μg) or polymyxin B (300 units) disks (Oxoid) following the methodology published by the European Society of Clinical Microbiology (EUCAST) (EUCAST, 2019). Zones of growth inhibition were measured in millimeters and sensitivity (S) determined based on the EUCAST guidelines. Broth microdilution assays were performed with vancomycin (Sigma) and the minimum inhibitory concentration (MIC, μg/ml) was determined according to the methodology published by Wiegand et al. (2008).

### *Galleria mellonella* Model of Infection

*Galleria mellonella* larvae were purchased from Livefoods Direct (United Kingdom). Bacterial cells harvested from 24 h plate cultures were re-suspended in PBS and OD_600_ adjusted to 0.1. Ten *G. mellonella* larvae per bacterial strain were injected with 10 μl bacterial suspension with a micro syringe (Hamilton, Switzerland) in the right foremost leg. Controls injected with PBS or not injected were also prepared. Larvae were incubated at 37°C, with counts of dead larvae recorded every 24 h for 5 days.

### Chicken Cell Interaction and Invasion Assays

Isolation of chicken intestinal primary cells (Byrne et al., 2007) as well as interaction and invasion assays (Corcionivoschi et al., 2009) were performed as described previously. Briefly, biopsies from sections of small intestines from 6 to 12 week-old broiler chickens (Cobb 500) were placed in primary cell culture medium and primary cells were isolated. *C. jejuni* strains were grown for 24 h on Muller-Hinton agar under microaerobic conditions. Bacterial cells were washed and resuspended in tissue culture medium to an OD_600_ of 0.4, then added to chicken intestinal primary cells grown in tissue culture plates to yield a multiplicity of infection of 1000:1. Plates were centrifuged and incubated for 3 h at 37°C in a microaerophilic environment. For interaction assays, infected monolayers were washed with PBS and treated with 0.1% v/v Triton X-100. Serial dilutions were performed and the CFUs/ml were enumerated. For invasion assays, infected monolayers were washed with PBS and treated with gentamicin (400 μg/ml) for 2 h at 37°C. Cells were then washed with PBS and treated with 0.1% v/v Triton X-100. Serial dilutions were performed and the CFUs/ml were enumerated.

### Chicken Infection Assay

Thirty male broiler chickens (Ross 308) were housed in isolation units on wood shaving bedding. The temperature in the isolation unit was kept between 22–25°C and thermostatically controlled. Broilers were fed *ad libitum* with a standard diet. *C. jejuni* strains were grown on Muller Hinton plates for 24 h under microaerobic conditions and resuspended in sterile distilled water. At 15 days old, ten broilers were inoculated with approximately 1 × 10^8^ CFU/ml of either the 488 wild-type strain, the 488 *tssD* mutant or the 81–176 wild-type strain. The different batches of infected broilers were kept separated in sterile isolation units. After 3 days of infection, broilers were euthanized and *C. jejuni* was enumerated by analyzing the cecum content using the ISO17025 methodology for plate counting. All broilers were confirmed using cloacal swabs as being *Campylobacter* free at the time of infection. These experiments were performed in triplicate on three separate occasions.

### Statistical Analysis

All experiments were performed with at least three biological replicates and three technical replicates, unless otherwise stated. Statistical analyses were performed using GraphPad Prism 7 (GraphPad Software, United States) and data were presented as mean ± SEM. Results were compared using unpaired *t*-test with ^∗^*p* ≤ 0.05, ^∗∗^*p* ≤ 0.01, ^∗∗∗^*p* ≤ 0.001.

## Data Availability Statement

The genome sequence datasets generated for this study can be found in EBI ENA, PRJEB31331.

## Ethics Statement

The experiments were performed according to the legislation in place (Law 471/2002 and government ordinance 437/2002) and under the supervision of National Sanitary Veterinary Agency. The Ethics Committee of Banat University of Agricultural Sciences and Veterinary Medicine – King Michael I of Romania approved this work.

## Author Contributions

OG and ND conceived the study with input from AH and NC. JL, GH, CD, AE, FS, AS, and OG performed the experiments and contributed to this manuscript. JL, OG, and ND wrote the manuscript with significant input from LS, IP, NC, and BW.

## Conflict of Interest

The authors declare that the research was conducted in the absence of any commercial or financial relationships that could be construed as a potential conflict of interest.
