# Supplementary material for: The Campylobacter jejuni Type VI Secretion System Enhances the Oxidative Stress Response and Host Colonization
Source: Front Microbiol. 2019 Dec 17;10:2864. doi: 10.3389/fmicb.2019.02864 (PMC6927950; doi:10.3389/fmicb.2019.02864)
Supplement: Supplementary file 1 [file Data_Sheet_1.PDF]

SUPPLEMENTARY INFORMATION

Table S1. *C. jejuni* 488 strain genome coordinates.

TSS System	Coordinates
**TssA**	**525818..527047**
**TssB**	**525264..525749**
**TssC**	**523808..525262**
**TssD**	**531562..532077**
**TssE**	**523413..523805**
**TssF**	**522589..523416**
**TssG**	**520972..521265**
**TssH**	**529563..530219**
**TssI**	**520821..519097**
**TssJ**	**527048..527473**
**TssK**	**527483..528880**
**TssL**	**528877..529683**
**TssM**	**530216..531526**

Table S2. Antimicrobial susceptibility testing.

**Antibiotics**	**488 wild-type**	**488 *tssD* mutant**	**488 *tssD* complement**
Ampicillin (10 µg)	S (31)	S (31)	S (31)
Amoxycillin/Clavulonic acid (30 µg)	S (30)	S (30)	S (30)
Tetracycline (30 µg)	S (40)	S (40)	S (40)
Polymyxin B (300 units)	S (20)	S (20)	S (20)
Vancomycin (MIC, µg/ml)	256	256	256

Antimicrobial susceptibility of 488 wild-type, 488 *tssD* mutant, and 488 *tssD* complement strains. The disk diffusion assay was performed with ampicillin (10 µg), amoxycillin/clavulonic acid (2:1, 30 µg), tetracycline (30 µg), and polymyxin B (300 units) disks (Oxoid) following method published by the European Society of Clinical Microbiology (EUCAST) [67]; zones of growth inhibition were measured in millimetres and sensitivity (S) determined based on EUCAST guidelines. Broth microdilution was performed with vancomycin (Sigma) and the minimum inhibitory concentration (MIC, µg/ml) was determined according to method published by Wiegand *et al.* [68]. The experiments were performed on three independent occasions.

Table S3. ***Campylobacter jejuni* strains used in this study.**

***C. jejuni* strains**	**Description**	**Source/Reference**
T6SS-positive		
488	Wild-type human isolate from Brazil	This study
488 *tssB*	488 *tssB::Cm*	This study
488 *tssC*	488 *tssC::Km*	This study
488 *tssBC*	488 *tssB::Cm tssC::Km*	This study
488 *tssD*	488 *tssD::Km*	This study
488 *tssD* complement	488 *tssD::Km* complemented with a copy of *tssD* in pRRC plasmid	This study
43431	Wild-type human isolate from Canada	[35]
T6SS-negative		
81-176	Wild-type human isolate from the United States	[69]

Table S4. ***Escherichia coli* strains used in this study.**

***E. coli* strains**	**Description**	**Source/Reference**
SCS110	Competent cells deficient in Dam and Dcm methylases	Agilent Technologies
XL2-Blue MRF	Competent cells for cloning methylated DNA	Agilent Technologies

Table S5. Primers used in this study.

Primer Name	Sequences	Source
Mutagenesis		
tssB F	TGACAAAGAACATACACTACAAG	This study
tssB R	AATCTAAGTCCACGCC	This study
tssB IPCRM F	GGGAGATCTGAACAGGAGCTGAAGAGC	This study
tssC IPCRM R	GGGAGATCTTTGTTGTTATATTGAGTTTTC	This study
tssC F	AAGCAGTTGATATGCC	This study
tssC R	AATGAGGTCGGACAC	This study
tssC IPCRM F	GGGAGATCTGACTGTTAAGGATCTGCCTAC	This study
tssC IPCRM R	GGGAGATCTTCCACACCATCTATATTTAGC	This study
tssD F	ATTGAAGGTTCCACACAAGG	This study
tssD R	GTTGATAATCTCCAAT	This study
tssD complement F	CCCTCTAGAATGAAGCGAGTAGATTTTGCCAAGCATTAAATA	This study
tssD complement R	CCCTCTAGATTAAATTCACGATACAATCC	This study
Kan^R F out	TGGGTTTCAAGCATTAGTCCATGCAAG	[48]
Kan^R R out	GTGGTATGACATTGCCTTCTGCG	[48]
Cam^R F out	CGATTGATGATCGTTGTA	[48]
Cam^R R out	TACAGCAGACTATACTG	[48]
RT-PCR		
tssB F	TGAGGATGTGGAGTTG	This study
tssB R	ATTACCCATAGGACCT	This study
tssC F	TTGATGAAATGATAGC	This study

tssC R	GTTGATAATCTCCAAT	This study
tssD F	TGAAGGTTCCACACAAGG	This study
tssD R	CACTTGTTGCGGTTCTAA	This study
qRT-PCR		
katA F	AACAAGCTGCCTTTAGTCCAAG	This study
katA R	CATAGCACCAGCGACATTGTAAG	This study
sodB F	GTGGCTGTGGCGGTTCATGTC	This study
sodB R	CTGCGTTTGAAGTACCTACA	This study
ahpC F	ATCAAGGTGGTATTGGTCAG	This study
ahpC R	TAACCACAGCATGGCGAACTG	This study
gyrA F	GTTATTATAGGTCGTGCTTT	This study
gyrA R	CTATGAGGTGGGATGTTTGT	This study

(IPCRM = Inverse PCR mutagenesis. Kan = Kanamycin. Cam = Chloramphenicol.)
